# Sweet Sorghum Genotypes Tolerant and Sensitive to Nitrogen Stress Select Distinct Root Endosphere and Rhizosphere Bacterial Communities

**DOI:** 10.3390/microorganisms9061329

**Published:** 2021-06-18

**Authors:** Lucas Dantas Lopes, Yen Ning Chai, Ellen L. Marsh, John F. Rajewski, Ismail Dweikat, Daniel P. Schachtman

**Affiliations:** Department of Agronomy and Horticulture, Center for Plant Science Innovation, University of Nebraska—Lincoln, Lincoln, NE 68588-0660, USA; ldantaslopes2@unl.edu (L.D.L.); yenningchai@huskers.unl.edu (Y.N.C.); emarsh2@unl.edu (E.L.M.); jrajewski1@unl.edu (J.F.R.); idweikat2@unl.edu (I.D.)

**Keywords:** microbial ecology, soil nitrogen, rhizobiome, agricultural microbiology, plant-microbe interactions

## Abstract

The belowground microbiomes have many beneficial functions that assist plant growth, including nutrient cycling, acquisition and transport, as well as alleviation of stresses caused by nutrient limitations such as nitrogen (N). Here we analyzed the root endosphere, rhizosphere and soil bacterial communities of seven sweet sorghum genotypes differing in sensitivity to N-stress. Sorghum genotypes were grown in fields with no (low-N) or sufficient (high-N) N. The dry shoot weight ratio (low-N/high-N) was used to determine N-stress sensitivity. Our hypothesis was that genotypes tolerant and sensitive to N-stress select distinct bacterial communities. The endosphere and rhizosphere bacterial community structure were significantly different between the N-stress sensitive and tolerant genotypes in the high-N field, but not in the low-N field. However, significant changes in the relative abundance of specific bacterial taxa were observed in both fields. *Streptomyces*, a bacterial genus known to alleviate plant abiotic stresses, was enriched in the endosphere and rhizosphere of the tolerant genotypes in the low-N field. Our study indicates that sweet sorghum genotypes tolerant to N-stress select taxa that can potentially mitigate the N-stress, suggesting that the interactions between N-stress tolerant lines and the root-associated microbiome might be vital for coping with N-stress.

## 1. Introduction

The collection of microbes inhabiting the soil in close contact with roots and directly influenced by rhizodeposition is known as the rhizosphere microbiome [1,2,3,4,5]. It harbors a multitude of microbes that can be neutral (commensals), beneficial (mutualists) or even harmful (pathogens) to the plant hosts [1,2]. Among the beneficial, there are multiple bacterial species able to promote plant growth and health through nutrient acquisition, phytohormones production, alleviation of biotic and abiotic stresses, etc. [1,2]. Plant-associated microbiomes have a significant impact on plant phenotypic plasticity and root function across varying environmental conditions [6,7,8,9]. The root-associated microbes are recruited from the bulk soil, which has a different microbial community structure and lower microbial activity/biomass than the rhizosphere [3,4]. Plants interact and shape the associated microbial communities by regulating root exudate profiles, root morphology and immune system [10,11]. Some microbes also colonize the root inner tissues, composing the root endosphere microbiome [1]. Many exudate compounds have been identified as important to shape the root-associated microbiome, including secondary metabolites, hormones, organic acids, and sugars [10]. Moreover, the soil physico-chemical factors and agricultural practices also have a huge contribution on the root microbiome assembly [5].

Soil nitrogen (N) availability is one of the most important environmental factors affecting plant performance and the associated microbiomes [12,13]. For example, increases in maize root exudation of phenolics, sugar alcohols, and sugars occurred after urea addition, resulting in an increased abundance of nitrifying and denitrifying bacteria in the rhizosphere [14]. Many plant-associated bacteria are important in improving plant fitness under stressful conditions [15]. For instance, the colonization of diazotrophs such as *Azospirillum* spp.*, Azoarcus* spp*.,* and *Herbaspirillum* spp. into the rhizosphere or endosphere were efficient in reducing plant N-stress by directly providing the plants with fixed N [16]. Understanding how plants shape bacterial communities to increase N-stress tolerance is essential for reducing the environmental impact of excess N fertilization due to agricultural practices. Such practices may cause leaching of N into water tables, N runoff into surface water and the emission of the greenhouse gas N_2_O through denitrification [17,18].

The effect of soil N availability on the bulk soil microbiome has been well-characterized in the last decades [19,20,21]. Some studies also analyzed the impact of different N-levels on root-associated microbiomes, such as *Medicago*, rice, sugarcane, wheat, and sorghum [13,22,23,24,25,26,27]. However, most studies focused on comparing plants growing under high-N versus low-N conditions, while the microbiome changes between different plant genotypes in the context of varying soil N levels has not been widely investigated. In sorghum, there is a huge variation in tolerance to N stress between genotypes [28]. Despite the fact that root-associated microbial communities of sorghum change between N-levels [24,26,27], the natural variability of the microbiome between N-stress tolerant and sensitive sorghum genotypes is unclear. In addition to physiological differences, it is possible that the higher fitness of the tolerant genotypes under low-N could be due to a specific microbiome selection, which could potentially help to mitigate the N-stress. Therefore, understanding the dynamics of the sweet sorghum belowground microbiomes under different soil N levels will be pivotal for increasing the productivity of this crop. Sweet sorghum also stores significant amounts of sugar within the stalk, so the optimization of this crop can potentially replace the conventional use of maize as a biofuel feedstock, whose priority should be for food instead of energy [29,30]. In addition, sorghum has a lower susceptibility to drought stress than maize as well as an enhanced N use efficiency [31,32].

Here we performed field experiments across two consecutive years (2014 and 2015) and used high-throughput next generation sequencing to assess changes in the rhizosphere, root endosphere, and soil bacterial communities of seven sweet sorghum genotypes differing in sensitivity to N-stress, which were grown in two fields with and without added N fertilization. We used the dry-weight ratio of plants grown in the low-N/high-N fields to determine the sensitivity of each sorghum genotype to N-stress [32]. Then we assessed the shifts in the bacterial communities of the three sorghum genotypes classified as N-stress tolerant compared to the four sorghum genotypes classified as N-stress sensitive. Our aims were to investigate (I) if sorghum genotypes tolerant to N-stress had a different bacterial community structure and diversity compared to N-stress sensitive genotypes, and (II) identify the bacterial taxa that were enriched in the N-stress tolerant genotypes, which may potentially be associated with the increased stress tolerance of these genotypes. Our study comprising three sample types (root, rhizosphere, and soil) collected from eight plant genotypes, cropped under two agronomic conditions (N-levels) and sampled in two growth stages in two different years, allowed a robust inference regarding the differential effect of sorghum genotypes tolerant or sensitive to N-stress on the root-associated bacterial communities.

## 2. Materials and Methods

### 2.1. Field Experiments

This study was conducted at the Eastern Nebraska Research and Extension Center (ENREC) located in Mead, Nebraska, USA during the summers of 2014 and 2015 under conventional tillage dryland non-irrigated conditions. Nine g m^−2^ of N in the form of anhydrous ammonia was applied early in the spring of each year to the high-N field (41.156414, −96.408031), which had a soybean-sorghum rotation history for the past 15 years. The low-N field (41.163166, −96.424108) had no N fertilizer amendment in the current experiments and had not received any fertilization in the past 15 years, during which time it was cropped with a sorghum-oat rotation with oat forage completely removed after each season.

The low-N and high-N fields were planted in a completely randomized design. Each plot in both fields had two rows containing one sorghum genotype with a distance of 76 cm between rows and approximately 45 plants in each 4 m-long row. Seven sweet sorghum genotypes were planted in the low-N and high-N fields with two replicates in 2014 and four replicates in 2015 (each plot represented a single replicate). The seven sorghum genotypes used were UNL3016 (Macia), Rancher, N108B, N109B, N110B, Northern Sugar Cane, Theis (Table 1). In addition, Nebraska Pearl Millet Hybrid-1 (NFPMH-1) was included in both years as a comparison with the sorghum genotypes (Table 1). Therefore, we collected 64 samples in 2014 (2 plots × 8 plant genotypes × 2 growth stages × 2 N-levels) and 128 samples in 2015 (4 plots × 8 plant genotypes × 2 growth stages × 2 N-levels) for both the rhizosphere and endosphere compartments. For soil, we collected 64 samples in 2014 (2 plots × 8 plant genotypes × 2 growth stages × 2 N-levels) and 40 samples in 2015 (10 composite samples for all plots and genotypes × 2 growth stages × 2 N-levels), generating a total of 488 samples in the study. The planting dates for each year were 30 May 2014, and 2 June 2015. Seeds were treated with CONCEP III (fluxofenim) prior to sowing to protect the seedlings from herbicide injury. In 2014 the rainfall during the time of the experiment (1 June to 15 September) was 436.88 mm, while in 2015 the rainfall during the time of the experiment (1 June to 29 September) was 473.71 mm.

### 2.2. Field Sample Collection

Samples from the seven sorghum genotypes and one pearl millet genotype cropped in both years were collected for 16S rRNA gene high-throughput sequencing. Soil, rhizosphere, and root endosphere samples were collected two times during the growing season. The first sampling was conducted during the plant vegetative stage, while the second sampling was performed when most genotypes were transitioning between vegetative and reproductive stage (flowering time differed between genotypes). In 2014, the samplings of the low-N field were on August 12 and September 15, whereas the samplings of the high-N field were on July 28 and September 29. In 2015, the sampling dates for the low-N field were on July 23 and September 28, whereas the high-N field was sampled on July 22 and September 23. The soil samples from different genotypes were combined in composite samples for each plant growth stage and N-level in 2015, since no microbiome differences between N-stress tolerant and sensitive genotypes were observed in soil based on 2014 data.

During the sampling, two plants per genotype were excavated from different random locations in each plot and roots were collected. The excess soil in close proximity to roots was shaken off, collected into a quart-size Ziploc bag and placed on ice. A variety of roots was collected including crown, seminal, and primary roots and placed in a 50 mL tube containing 35 mL of phosphate buffer (6.33 g L^−1^ NaH_2_PO_4_, 8.5 g L^−1^ Na_2_HPO_4_ anhydrous, 200 µL L^−1^ Silwet L-77). Tubes containing the root samples were vigorously shaken for 30 to 60 s to wash off rhizosphere soil from the roots. The roots were then removed, blotted briefly, and placed in a clean 50 mL tube. Both tubes containing excised roots and rhizosphere soil with phosphate buffer were placed on ice.

Roots were surface sterilized by rinsing for 30 s in 5.25% sodium hypochlorite and 0.01% Tween 20, followed by a 30 s rinse in 70% ethanol, and three rinses in sterile ultrapure water. Roots were blotted dry on a clean paper towel, placed in a 15 mL tube, and frozen at −80 °C prior to being ground in liquid N for DNA extraction. The rhizosphere samples were filtered through a sterile 100 µm mesh filter unit (Fisher Scientific, Waltham, MA, USA) into a sterile 50 mL tube. The rhizosphere was pelleted at 3000× *g* for 10 min at room temperature using a centrifuge. The pellet was resuspended in 1.5 mL phosphate buffer (6.33 g L^−1^ NaH_2_PO_4_, 8.5 g L^−1^ Na_2_HPO_4_ anhydrous), and transferred to a sterile 2 mL microcentrifuge tube. The rhizosphere was re-pelleted by spinning tubes for 5 min at full speed. The supernatant was discarded, and the rhizosphere pellet was stored at −20 °C until DNA extraction. Soil was sieved through US Standard Sieve #4 (4750 microns), followed by Sieve #8 (2360 micron) to remove debris and roots, and stored at −20 °C prior to DNA extraction. More sampling details are found in McPherson et al., 2018 [33].

### 2.3. Plant Phenotyping

Plant height, flowering time, stalk moisture, fresh and dry biomass were measured for all genotypes grown in the high and low-N fields in both years. Dry weight ratio obtained by dividing the plant dry weight grown in the low-N field by the plant dry weight grown in the high-N field was used to determine the susceptibility of each genotype to N-stress [32]. The smaller the ratio, the more sensitive the genotype is to N-stress and vice-versa. Using this ratio, we classified all genotypes as N-stress sensitive or tolerant (Table 1). The 2015 data were used for this classification because the N-stress was too severe in 2014, leading to very low biomass in all the genotypes grown under N-stress.

### 2.4. DNA Extraction, 16S rRNA Gene Amplification, and High-Throughput Sequencing

DNA was extracted from soil and rhizosphere samples using the PowerSoil-htp 96 Well Soil DNA Isolation Kit (MoBio, Carlsbad, CA, USA). Root endosphere DNA was extracted with PowerPlant Pro-htp 96 Well DNA Isolation Kit (MoBio, Carlsbad, CA, USA) following manufacturers protocol. The DNA was quantified with Quantifluor dsDNA (Promega) following manufacturers guidelines.

The 16S rRNA amplicons were sequenced at the University of Minnesota Genomics facility using universal primers 515F and 806R targeting the V4 hypervariable region [34]. For DNA samples collected from the 2014 experiment, sequencing was done using paired-end 250 base reads on an Illumina HiSeq platform. For DNA samples collected from the 2015 experiment, sequencing was done using paired-end 300 base reads on an Illumina MiSeq platform. The same amplification protocols were used in each platform with the inclusion of peptide nucleic acid (PNA) blockers in the root DNA samples during PCR, to reduce the amount of chloroplast and mitochondrial 16S rRNA amplicon contamination.

### 2.5. Bioinformatics and Statistical Analyses

The raw sequencing reads were de-multiplexed, merged, trimmed, filtered, and clustered into operational taxonomic units (OTUs) with a cutoff of 97% similarity using UPARSE [35]. The taxonomy assignment was performed using the RDP classifier trained on the Greengene database implemented in QIIME 1.9.1 with a minimum confidence score of 0.80 [36]. The reads classified as mitochondria and chloroplast were discarded prior to further processing. To account for the difference in sequencing sampling depth, samples from different compartments (soil, rhizosphere, and root) were analyzed separately and rarefied to reach a minimum Good’s coverage score of 0.90. Shannon-Wiener and Chao1 indices were used to estimate the species diversity and richness in the samples, respectively [37,38]. The Kruskal-Wallis and Wilcoxon tests were used to detect differences in α-diversity using QIIME 1.9.1 software [36].

In order to analyze shifts in bacterial community structure associated with the factors assessed in our study (N-level, genotype, tolerance to N-stress, growth stage, and year), non-metric multidimensional scaling (NMDS) and canonical analysis of principal coordinates (CAP) were performed using the Bray-Curtis distance matrices from the OTU tables with *metaMDS* and *capscale* functions implemented in the “*vegan”* package in R software (version 2.5-6) [39]. NMDS was used for showing the major factors that shaped microbial community structure (N-level, growth stage and year), while CAP was used to show the changes associated with more specific factors (plant genotype and N-stress sensitivity class). In the CAP models, we conditioned those major factors to remove their effects on the analysis of specific factors. The resulting plots were visualized using *ggplot* function in the “*ggplot2”* package (version 3.3.0) [40]. In addition, multivariate tests of hypotheses were performed with permutative multivariate analysis of variance (PERMANOVA, 999 permutations) using the function *anova* and *adonis* in “*vegan*” package to assess the effect of each factor and their interactions in shaping the bacterial communities.

Linear discriminant analysis (LDA) effect size (LEfSe) method was used to detect the microbial taxa with significantly different relative abundances between the N-stress sensitive and tolerant lines planted in both fields [41]. A *t*-test was used to assess the differences in the dry-weight ratio between the N-stress tolerant and sensitive genotypes, while one-way ANOVA and Tukey test were used to assess differences in plant biomass (dry weight) between genotypes, high versus low-N fields, and N-stress tolerant versus sensitive sorghum lines using the R software.

## 3. Results

### 3.1. Differences in Plant Biomass between Genotypes, N-Levels, and N-Stress Sensitivity Classes

**Figure 1:**
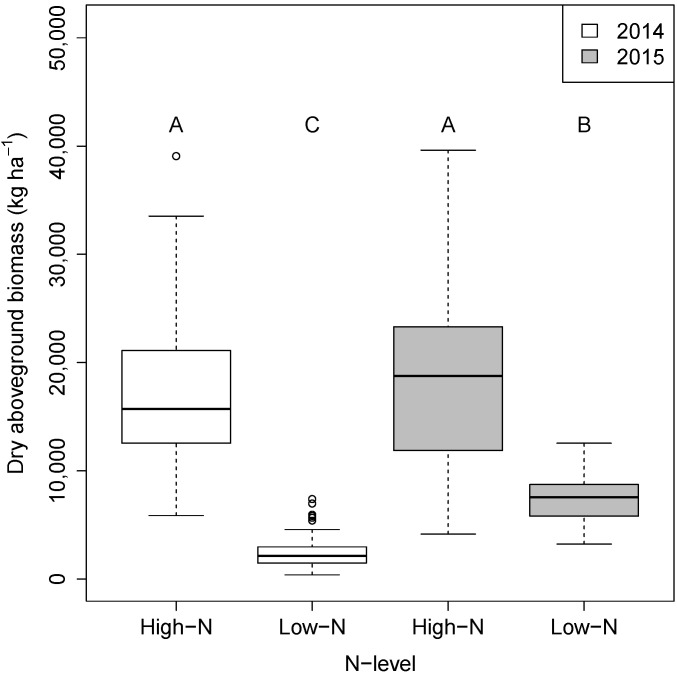
Changes in dry aboveground biomass between plants grown at different N levels. Box plots of the dry aboveground biomass (Kg ha^−1^) of all genotypes planted in the high- and low-N fields in 2014 and 2015. Different letters indicate significant differences according to Tukey test (*p* < 0.05).

The plants grown in the low-N field flowered later and had significantly lower dry weight compared to those grown in the high-N field regardless of genotype or year (Figure 1). Shoot biomass (dry weight) was 7.1 and 2.4 times greater in the high-N compared to the low-N field in 2014 and 2015, respectively (Figure 1). Overall, the shoot biomass of the plants grown in 2014 was significantly lower than those in 2015 in the low-N fields (Figure 1), but was not significantly different between years in the high-N field. The low biomass of all genotypes in the low-N field in 2014 was due to a high degree of stress which resulted in very little differentiation between genotypes. Therefore, we used the dry weight ratio (high-N/low-N) data from 2015 to categorize the genotypes into N-stress tolerant and sensitive classes. The pearl millet genotype (NFPMH-1) and four sorghum genotypes (N108B, N110B, Theis and Northern Sugarcane) had significantly lower dry weight ratios (*p* < 0.01) and were therefore classified as being more N-stress sensitive than the other three sorghum genotypes (Macia, N109B and Rancher), which were classified as being more N-stress tolerant (Table 1).

### 3.2. Changes in α and β-Diversity between Compartments, N-Levels, Growth Stages and Years

**Figure 2:**
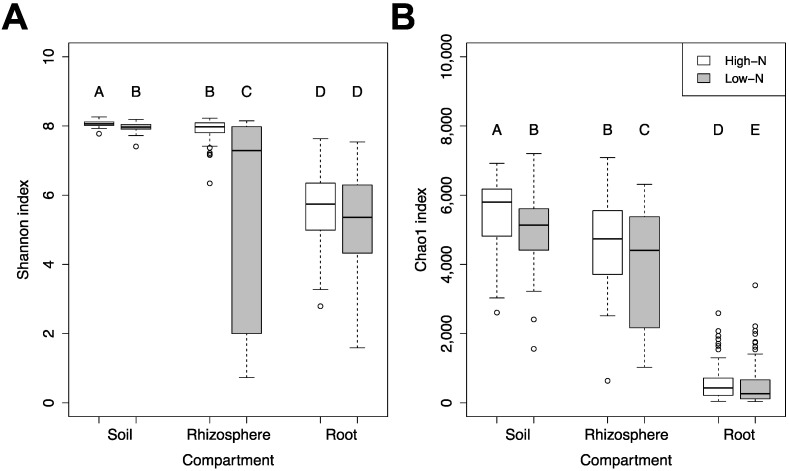
Differences in *α*-diversity between the three compartments and two N-levels. Box plots of *α*-diversity at two N-levels in soil, rhizosphere, and root endosphere compartments in both years sampled. (**A**) Differences in species diversity calculated by the Shannon index; and (**B**) differences in species richness calculated by the Chao1 index. Different letters indicate significant differences according to Wilcoxon’s pairwise test.

The bacterial community α-diversity differed between soil, rhizosphere, and root endosphere (Figure 2). Both species diversity and richness significantly decreased in the endosphere compared to the rhizosphere and soil compartments (Figure 2). Species diversity and richness were significantly higher under high-N as compared to low-N in the soil and rhizosphere compartments, while only species richness was significantly higher under high-N in the root endosphere (Figure 2). When comparing soil to rhizosphere, both α-diversity indices were significantly higher in soil than in rhizosphere within each N-level (Figure 2). No differences in species diversity and richness were found between the early and late growth stages in either year in the soil compartment (Figure S1A). In the rhizosphere compartment, α-diversity (species richness and diversity) increased later in the season in both years (Figure S1B). On the other hand, in the root endosphere species richness was higher in the later growth stage, but species diversity was not different between growth stages (Figure S1C).

**Figure 3:**
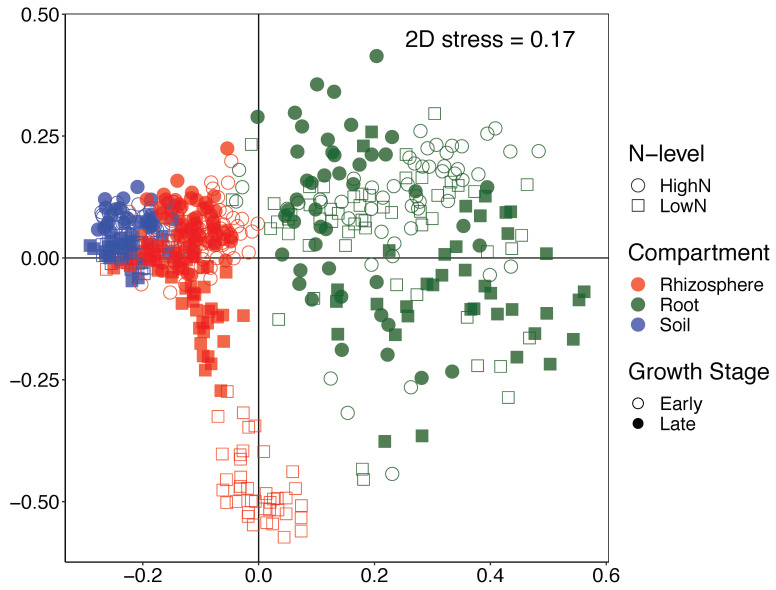
Shifts in β-diversity between soil, rhizosphere, and root endosphere bacterial communities. Non-metric multidimensional scaling (NMDS) based on the Bray-Curtis distance matrix considering all samples in the study including root, rhizosphere and soil, N-levels, and plant growth-stages. Stress value is shown on the graph.

The β-diversity was also changed due to the factors analyzed in our study. A multivariate permutative analysis of variance (PERMANOVA) showed significant differences between compartments, N-levels, years, and growth stages, including significant interactions among all these factors (*p* < 0.001). PERMANOVA also revealed that the largest effect size on the bacterial communities was due to compartment (R^2^ = 0.132), followed by N-level (R^2^ = 0.030), growth stage (R^2^ = 0.022) and year (R^2^ = 0.017). A non-metric multidimensional scaling (NMDS) grouped the samples according to compartment, since this was the dominant factor influencing bacterial community structure (Figure 3).

**Figure 4:**
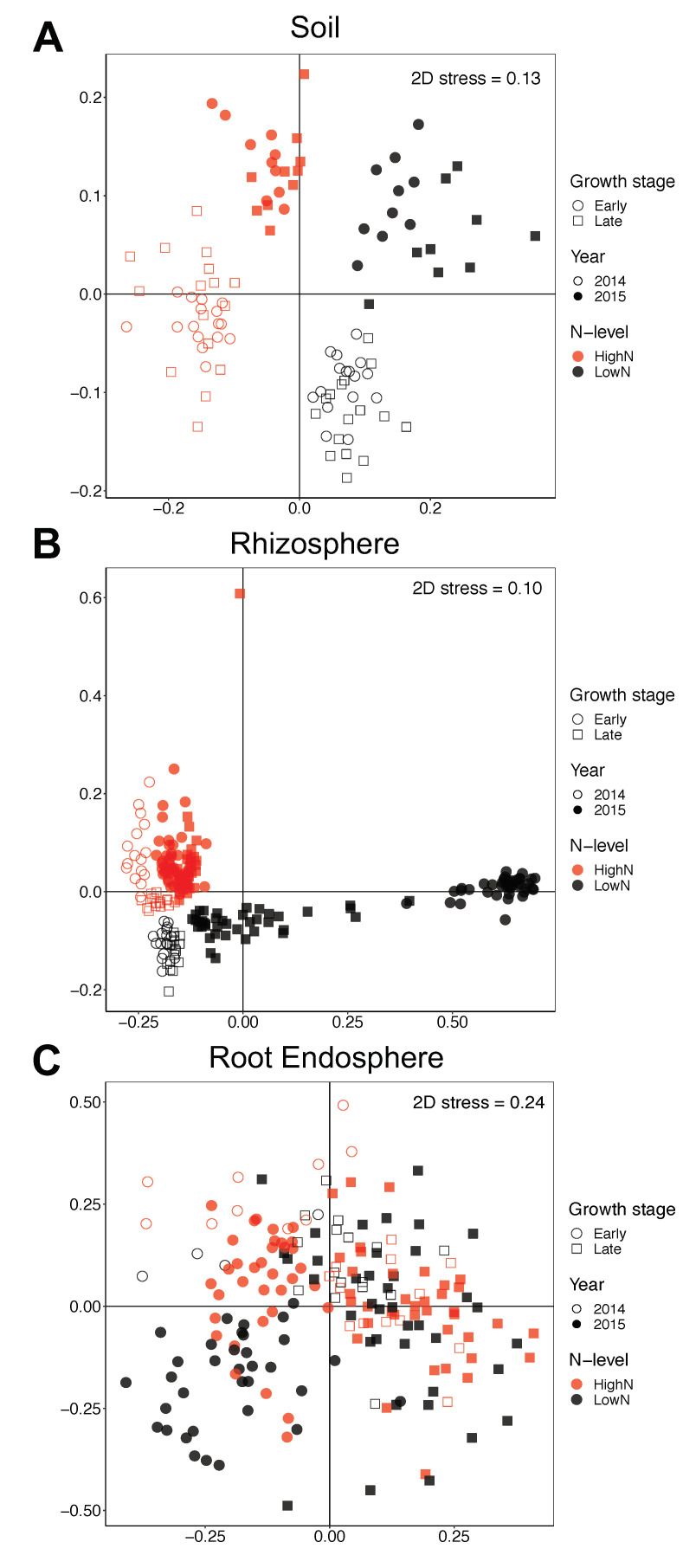
Changes in β-diversity showing the main factors influencing bacterial community structure in each compartment. Non-metric multidimensional scaling (NMDS) based on the Bray-Curtis distance matrices for (**A**) soil, (**B**) rhizosphere, and (**C**) root endosphere. Samples from different N-levels, plant growth stages and years of sampling are shown. Stress values are shown on each graph.

In order to better understand the effect of the other factors on the bacterial communities, we split the analysis by compartment. Growth stages, N-levels and years were all significant for each of the three compartments according to PERMANOVA (*p* < 0.001), but had different effect sizes in soil, rhizosphere, and endosphere. In the soil compartment, N-level was the most important factor (R^2^ = 0.164) affecting bacterial community structure, followed by year (R^2^ = 0.110) and growth stage (R^2^ = 0.035) (Figure 4A). The rhizosphere bacterial community structure was also more affected by the N-levels (R^2^ = 0.200), whereas growth stage (R^2^ = 0.117) had a greater effect than year (R^2^ = 0.103) in this compartment (Figure 4B). For the root endosphere, growth stage (R^2^ = 0.073) had the largest effect size, followed by N-levels (R^2^ = 0.034) and years (R^2^ = 0.020) (Figure 4C). It is noteworthy that growth stage had an increasingly larger effect on the bacterial communities as the contact with plant tissues increased (root endosphere > rhizosphere > soil), while the opposite trend was observed for the influence of years. On the other hand, the effect of N-levels was greater in the rhizosphere among the three compartments.

### 3.3. Shifts in Bacterial Community Structure and Composition between N-Stress Tolerant and Sensitive Genotypes

We tested next whether genotypes tolerant to N-stress showed differences in the soil and root-associated bacterial communities compared to the sensitive ones. Firstly, we assessed if there were differences in bacterial communities between all genotypes. For that, the pearl millet hybrid was removed from the analysis and only the seven sorghum genotypes were considered. The other factors (N-level, year, and growth stage) were conditioned to focus only on the effect of sorghum genotypes. PERMANOVA indicated that the sweet sorghum bacterial communities were significantly different between the genotypes in the soil (*p* < 0.01) and root endosphere (*p* < 0.001) compartments based on the canonical analysis of principal coordinates (CAP) (Supplementary Figure S2A,B). However, in the rhizosphere compartment there were only significant differences (*p* < 0.05) between the genotypes in 2014 (Figure S2C,D). In addition, there was a *Pseudomonas* spp. bloom in the rhizosphere at the early-stage sampling in 2015 under low-N (Figure S3A). This *Pseudomonas* spp. bloom was observed in the rhizosphere of all plant genotypes in the low-N field at the early stage in 2015 (Figure S3B). Therefore, the overwhelming dominance of *Pseudomonas* spp. in all rhizosphere samples in the low-N field at the early stage was the reason for the lack of significant differences in the rhizosphere bacterial community structure among the genotypes in 2015. The *Pseudomonas* spp. abundance decreased in the rhizosphere samples from plants in the later stage of sampling under low-N (2015) but remained the most abundant genus in all genotypes (Figure S3B).

**Figure 5:**
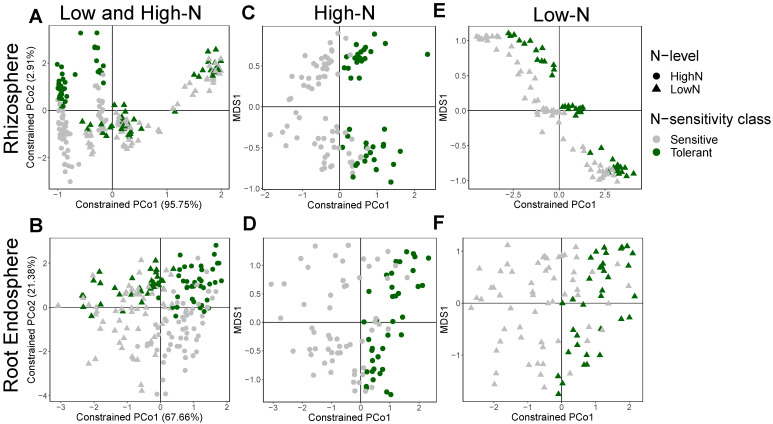
β-diversity differences between N-stress tolerant and sensitive genotypes. Canonical analysis of principal coordinates (CAP) based on the Bray-Curtis distance matrices considering samples from both N-levels in the (**A**) rhizosphere, and (**B**) root endosphere, as well as samples from only the high-N field in the (**C**) rhizosphere, and (**D**) root endosphere, and samples from only the low-N field in the (**E**) rhizosphere, and (**F**) root endosphere. Soil N-levels are represented with different symbols, while sorghum genotype N-stress sensitivity classes are represented with different colors.

Next, we assessed the differences between the N-stress tolerant versus sensitive genotypes. PERMANOVA and CAP showed that the root endosphere bacterial community structure was significantly different (*p* = 0.003; R^2^ = 0.010) between the N-stress tolerant and sensitive genotypes, but no significant differences were observed in the rhizosphere (Figure 5A,B). However, a separation of N-stress sensitive and tolerant genotypes was observed along axis 2 for the rhizosphere bacterial communities of plants grown on the high-N field (Figure 5A). Therefore, we split the data from the high-N and low-N fields to check the differences between N-stress sensitive and tolerant genotypes under each N-level. Both the rhizosphere (*p* = 0.006; R^2^ = 0.017) and root endosphere (*p* = 0.006; R^2^ = 0.019) bacterial communities were significantly different between N-stress sensitive and tolerant genotypes under high-N (Figure 5C,D), but none of them were significantly different in the low-N field, although both showed some separation along axis 1 (Figure 5E,F). In the soil compartment, no significant differences were observed between the N-stress tolerant and sensitive genotypes when analyzing samples from the high and low-N fields together (Figure S4A), nor when separating the data by N-level (Figure S4B,C), despite some separation of samples being observed. Moreover, no significant changes in α-diversity were observed between the N-stress sensitive and tolerant genotypes in any of the three compartments.

**Figure 6:**
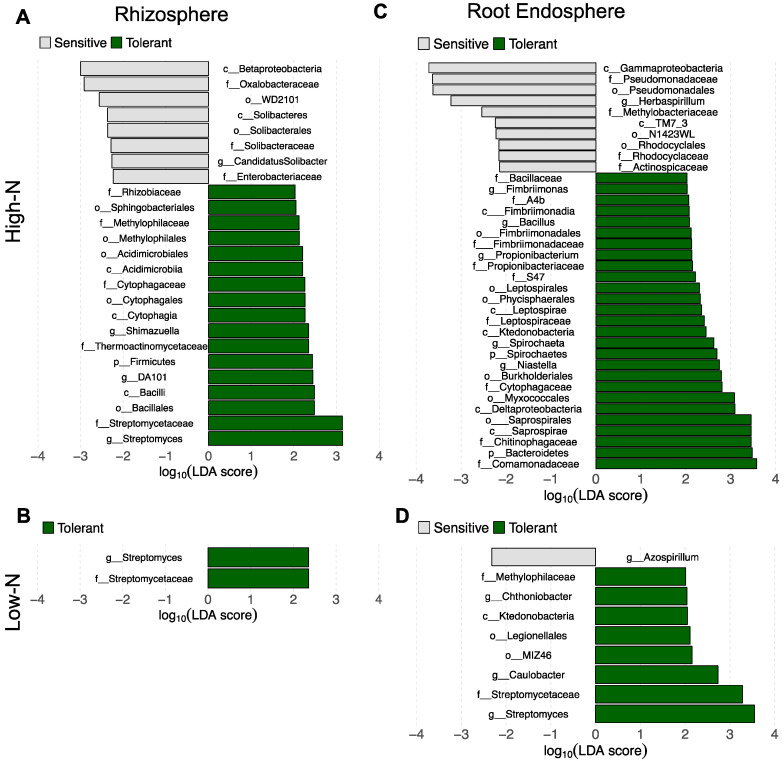
Differences in relative abundance of specific bacterial taxa. Taxa changing in relative abundance as determined by linear discriminant analysis (LDA, *p* < 0.05) between N-stress sensitive and tolerant sorghum genotypes in the (**A**) rhizosphere samples in the high-N field; (**B**) rhizosphere samples in the low-N field; (**C**) root endosphere samples in the high-N field; and (**D**) root endosphere samples in the low-N field.

Finally, we investigated changes in the relative abundance of specific bacterial taxa between the sorghum genotypes categorized as tolerant or sensitive to N-stress for each N-level. The comparison between the N-stress sensitive and tolerant genotypes in the rhizosphere compartment showed that 25 taxa changed in relative abundance in the high-N field and only two taxa changed in relative abundance in the low-N field (Figure 6A,B). In the high-N field, eight taxa were enriched in the sensitive genotypes including the family Enterobacteriaceae, while 17 taxa were enriched in the tolerant genotypes including the families Rhizobiaceae, Streptomycetaceae and the genus *Streptomyces* (Figure 6A). In the low-N field, only the family Streptomycetaceae and the genus *Streptomyces* were enriched in the rhizosphere of the tolerant genotypes (Figure 6B). In the root endosphere we also observed a higher number of taxa that differed significantly in relative abundance between N-stress tolerant and sensitive genotypes in the high-N field (37 taxa) than in the low-N field (nine taxa) (Figure 6C,D). In the high-N field, 10 taxa were enriched in the sensitive lines including the family Pseudomonadaceae and genus *Herbaspirillum*, while 27 taxa were enriched in the tolerant lines including the families Chitinophagaceae, Bacillaceae and the genus *Bacillus* (Figure 6C). In the low-N field, only the genus *Azospirillum* was enriched in the sensitive lines, while eight taxa were enriched in the tolerant lines including the family Streptomycetaceae and the genera *Caulobacter* and *Streptomyces* (Figure 6D).

## 4. Discussion

Our study confirmed previous work showing the significant impact of N fertilization levels on soil, rhizosphere, and root endosphere microbiomes. Soil N content is known to be an important environmental component affecting the composition, diversity, functionality, and biomass of soil microbial communities [12,19,20,21]. The changes in the soil microbiome due to N levels have been shown to associate with carbon (C) dynamics in many systems, since the dominant heterotrophic microbiota use plant residues and root exudates as nutritional sources [42,43], and microbial utilization of plant-derived organic compounds is influenced by N availability in the soil [12,44]. Different N fertilization levels affect the soil microbial communities of many important agricultural crops, such as wheat, maize, and sugarcane [20,45,46]. Our present study indicated that the soil bacterial community of sweet sorghum is also affected by different N inputs. In addition to changes in the soil microbiome, previous work showed a significant effect of N fertilization on the rhizosphere microbiomes of *Medicago*, wheat, maize, and sorghum [13,14,22,24,27,47], and in the root endosphere microbiomes of wheat, sorghum, rice and sugarcane [13,23,25,26]. These studies are largely in agreement with our results, since we also observed significant changes in the β-diversity of root endosphere and rhizosphere bacterial communities of plants growing under different N fertilization levels. However, we acknowledge that other factors such as different history of cropping and changes in other soil physico-chemical variables between the high-N and low-N fields used in our study may have had an impact on the differences between the bacterial communities associated with the plants growing in the two different fields.

Nevertheless, this study expanded the findings of previous work by analyzing seven different sweet sorghum genotypes with contrasting susceptibility to N-stress and comparing the effect of N-levels to other factors, such as growth stage and sampling year—which also affected the bacterial community structure in the three compartments. The influence of different N-levels was greater than growth stage and year in the soil and rhizosphere possibly because both compartments are in direct contact with soil N. On the other hand, the influence of N-levels was lower than that of growth stage in the root endosphere bacterial community probably because this compartment is more influenced by the host physiological factors than the soil and rhizosphere [13,48,49]. However, soil N content may alter root exudation processes and plant immunity [27], which in turn strongly impacts the microbes recruited to the endosphere microbiome [50]. In contrast to the weaker annual variation in the rhizosphere and endosphere bacterial communities, year of sampling had a more pronounced effect on the soil bacterial community, which is consistent with the large temporal differences observed in soil microbial communities in previous studies [51,52]. The increased influence of time and decreased influence of growth stage in the soil compartment might be due to a lower impact of host plant factors such as roots exudates in the non-rhizosphere soil bacterial community.

We also showed significant effects of different sorghum genotypes on the soil, rhizosphere and root endosphere bacterial communities. Genotypic effects on the rhizosphere microbiome have been reported in many plants, including maize, potato, wheat, rice, *Arabidopsis thaliana*, *Pinus radiata*, and *Populus trichocarpa* [53,54,55,56,57,58]. The intra-specific variation in the rhizosphere microbiome of sorghum was also shown in previous work analyzing two and five genotypes [59,60], which is consistent with our results. In addition to rhizosphere, our study showed that the sorghum root endosphere bacterial community shifts according to different genotypes, as observed in some other plant species [55,56]. Furthermore, we showed a significant effect of sorghum genotype on the soil bacterial community, which was reported in a single study with the tree *Populus angustifolia* [61], indicating that the plant intra-specific genotypic variability affects the soil microbial communities living beyond the rhizosphere. Investigation of the plant genotypic effect on the soil microbiome has lagged behind because most studies on this topic focused on root-associated microbial communities.

However, the most remarkable result in our study was that the root endosphere and rhizosphere bacterial communities were significantly different between the N-stress sensitive and tolerant genotypes (Figure 5), whereas this effect was not observed in the soil bacterial community (Figure S4). A previous study reported shifts in the rhizosphere microbiome between two maize genotypes with different N use efficiencies [62], but differences between genotypes sensitive or tolerant to low N have not previously been identified. The experimental design with multiple genotypes and two nitrogen treatments allowed for this observation, that suggests commonalities in the selection of specific bacteria among the N-stress tolerant genotypes, which is different to the selection performed by the N-stress sensitive genotypes used in this study. This differentiation of the bacterial communities may be associated with similar physiological responses to N availability among genotypes of the same category (tolerant or sensitive) and potentially contribute to the differential tolerance to N-stress.

Our results showed strong differences in the bacterial community structure and in the relative abundance of many bacterial taxa between N-stress tolerant and sensitive genotypes under high-N both in the root endosphere and rhizosphere compartments (Figure 5C,D and Figure 6A,B). On the other hand, under low-N the differences on the root endosphere and rhizosphere bacterial composition between N-stress sensitive and tolerant genotypes shrank, with no significant differences in community structure and less differences in the relative abundance of specific bacterial taxa. This shrinkage in differences was possibly associated with the N availability, which eliminated the bacteria that were less resilient to N-stress conditions. However, it is noteworthy that there was an enrichment of the Streptomycetacae family and *Streptomyces* genus in the tolerant genotypes in both rhizosphere and endosphere compartments under low-N, as well as in the rhizosphere under high-N (Figure 6). Many strains of the actinobacterial genus *Streptomyces* were shown to alleviate several types of abiotic stress to plants, such as saline, drought and heavy metals [63,64,65,66]. Therefore, our results suggest that under N-stress the tolerant genotypes select for *Streptomyces*, and we hypothesize that the higher tolerance of those genotypes to N stress might also be associated with the higher relative abundance of this genus. Further studies are needed to test the potential beneficial effects of *Streptomyces* to sweet sorghum under N-stress. If effective, different strategies such as *Streptomyces* inoculation or plant breeding directed to produce genotypes that naturally recruit the bacteria of this genus could be used to enhance sweet sorghum productivity and reduce the use of N chemical fertilizer.

In addition to *Streptomyces*, the root endosphere of the tolerant lines under low-N was also enriched in the genus *Caulobacter*, which has been shown to promote plant growth by unknown mechanisms [67]. Under high-N the tolerant lines were also enriched in other plant beneficial bacteria, such as *Bacillus* and Chitinophagaceae in the root endosphere [68,69], and Rhizobiaceae in the rhizosphere [70]. However, some plant beneficial bacteria were enriched in the sensitive lines under high-N, such as Pseudomonadaceae and Rhodocyclaceae in the root endosphere [71,72,73,74], and Enterobacteriaceae in the rhizosphere [75,76,77,78,79]. Interestingly, the endophytic N-fixing genera *Herbaspirillum* and *Azospirillum* were enriched in the root endosphere of the sensitive lines under high-N and low-N conditions, respectively [80,81,82]. These results are surprising because they suggest that N-stress sensitive lines host diazotrophic bacteria in root tissues which could compensate for the higher susceptibility to N starvation, while N-stress tolerant genotypes harbor bacteria able to alleviate the abiotic stress — which may be more effective. Therefore, this putative bacterial community selection strategy of different genotypes may be involved in the tolerance to N-stress or may just be an outcome of the stress levels in the different genotypes.

We observed a very large relative abundance of *Pseudomonas* spp. in the rhizosphere samples from all genotypes under low-N in the early plant growth stage in 2015. This bloom of *Pseudomonas* spp. was not observed in the soil and root compartments. A similar *Pseudomonas* spp. bloom was also previously reported in the maize rhizosphere, but not in soil [83]. In addition, a previous study using the same fields as ours but with different sorghum lines, also noted the *Pseudomonas* spp. bloom in the sorghum rhizosphere under low-N in the same period [27]. These results indicate that the *Pseudomonas* spp. bloom was probably a plant-driven phenomenon because it was found in the rhizosphere and not the soil bacterial community. Moreover, the observation of the bloom only in the low-N field suggests that it may have been triggered as a response to N stress. Analysis of the sorghum root metabolomics showed a negative correlation between the *Pseudomonas* spp. abundance with the defense hormone salicylic acid, suggesting the bloom was caused by a reduced defense response due to a compromised immune system of the plants under low-N [27]. *Pseudomonas* spp. are also known to associate with several plant species and to support plant growth and health [72,73,74]. Moreover, previous studies detected different populations and ecotypes of fluorescent *Pseudomonas* spp. in the sugarcane rhizosphere compared to bulk soil, suggesting selection by the host plants [84,85]. Therefore, one hypothesis is that the *Pseudomonas* bloom is a plant-driven phenomenon that may occur to help plants deal with the stressful conditions of N deprivation. Alternatively, the bloom may have no functional significance and may just be a consequence of suppression of the plants immune system by low-N conditions. Future studies are needed to clarify the reasons for the bloom, as well as the role of *Pseudomonas* spp. for N-limited plants. 

## 5. Conclusions

Our study showed that the sweet sorghum genotypic variation associated to N-stress tolerance affects the root endosphere and rhizosphere bacterial communities. The differences between the bacterial communities of N-stress tolerant and sensitive genotypes were most obvious under high soil N. However, under low soil N the differences were more restricted to specific bacterial taxa that we hypothesize might help the tolerant genotypes to cope with the N-stress, e.g., *Streptomyces*. These results advance the understanding of the impact that soil N availability has on the root-associated microbiomes; indicates a distinctive effect of sorghum genotypes on the belowground bacterial communities; and provides reference taxa for future studies aiming to experimentally test the effect of specific bacteria on sweet sorghum under N-stress. Overall, these findings contribute to new knowledge that could be harnessed towards the development of more sustainable agricultural systems that are less dependent on the input of massive amounts of inorganic N.

## Figures and Tables

**Table 1:** Plant genotypes used in this study. Shoot dry weight ratio of each genotype measured in 2015. The division of lines sensitive and tolerant to nitrogen (N) stress is shown. Different letters indicate significant differences according to *t*-test (*p* < 0.01).

Class	Genotype	Dry Weight Ratio (Low-N/High-N)
Sensitive	Forage PM Hybrid	0.3 _b_
	N108B	0.4 _b_
	N110B	0.4 _b_
	Theis	0.3 _b_
	Northern Sugarcane	0.4 _b_
Tolerant	Macia	0.5 _a_
	N109B	0.6 _a_
	Rancher	0.5 _a_

## Data Availability

The sequences used in this manuscript were submitted to NCBI-SRA under the BioProject accession number PRJNA693304.

## Supplementary Materials

The following are available online at https://www.mdpi.com/article/10.3390/microorganisms9061329/s1, Figure S1: α-diversity differences between years and plant growth stages. Box plots of the species diversity calculated with Shannon index on left and species richness calculated by Chao 1 index on right between the two growth stages and years for the (A) soil, (B) rhizosphere and (C) root endosphere compartments. Distinct letters indicate significant differences according to Wilcoxon’s pairwise test. Figure S2: β-diversity differences between sweet sorghum genotypes. Canonical analysis of principal coordinates (CAP) based on the Bray-Curtis distance matrices showing samples of the different sorghum genotypes from (A) root endosphere 2014 and 2015, (B) soil 2014, (C) rhizosphere 2014, and (D) rhizosphere 2015. All other factors except genotypes were conditioned in the model. Figure S3: Bar chart showing the relative abundance of bacterial families. (A) Relative abundance of the top 50 most abundant bacterial families in the root endosphere, rhizosphere and soil compartments in 2014 and 2015 separated by growth stage and N-level. (B) Rhizosphere samples from 2015 highlighting the very high abundance of *Pseudomonas* spp. in all genotypes at the early plant growth stage under low-N. Pseudomonadaceae family is shown in blue bar. The families found in the lowest abundance were combined into “other”. Figure S4: Bacterial community structure of soil samples from N-stress tolerant and sensitive genotypes. Canonical analysis of principal coordinates (CAP) based on the Bray-Curtis distance matrices considering soil samples from (A) both N-levels, (B) only the high-N field, and (C) only the low-N field. Soil N-levels are represented with different symbols, while sorghum genotype N-stress sensitivity classes are represented with different colors. Only samples from 2014 were used in the analysis, since samples from different genotypes were bulked in 2015.
