# Supplementary material for: Sweet Sorghum Genotypes Tolerant and Sensitive to Nitrogen Stress Select Distinct Root Endosphere and Rhizosphere Bacterial Communities
Source: Microorganisms. 2021 Jun 18;9(6):1329. doi: 10.3390/microorganisms9061329 (PMC8234256; doi:10.3390/microorganisms9061329)
Supplement: Supplementary file 1 [file microorganisms-09-01329-s001.zip › microorganisms-1256988-supplementary.pdf]

Supplementary Materials

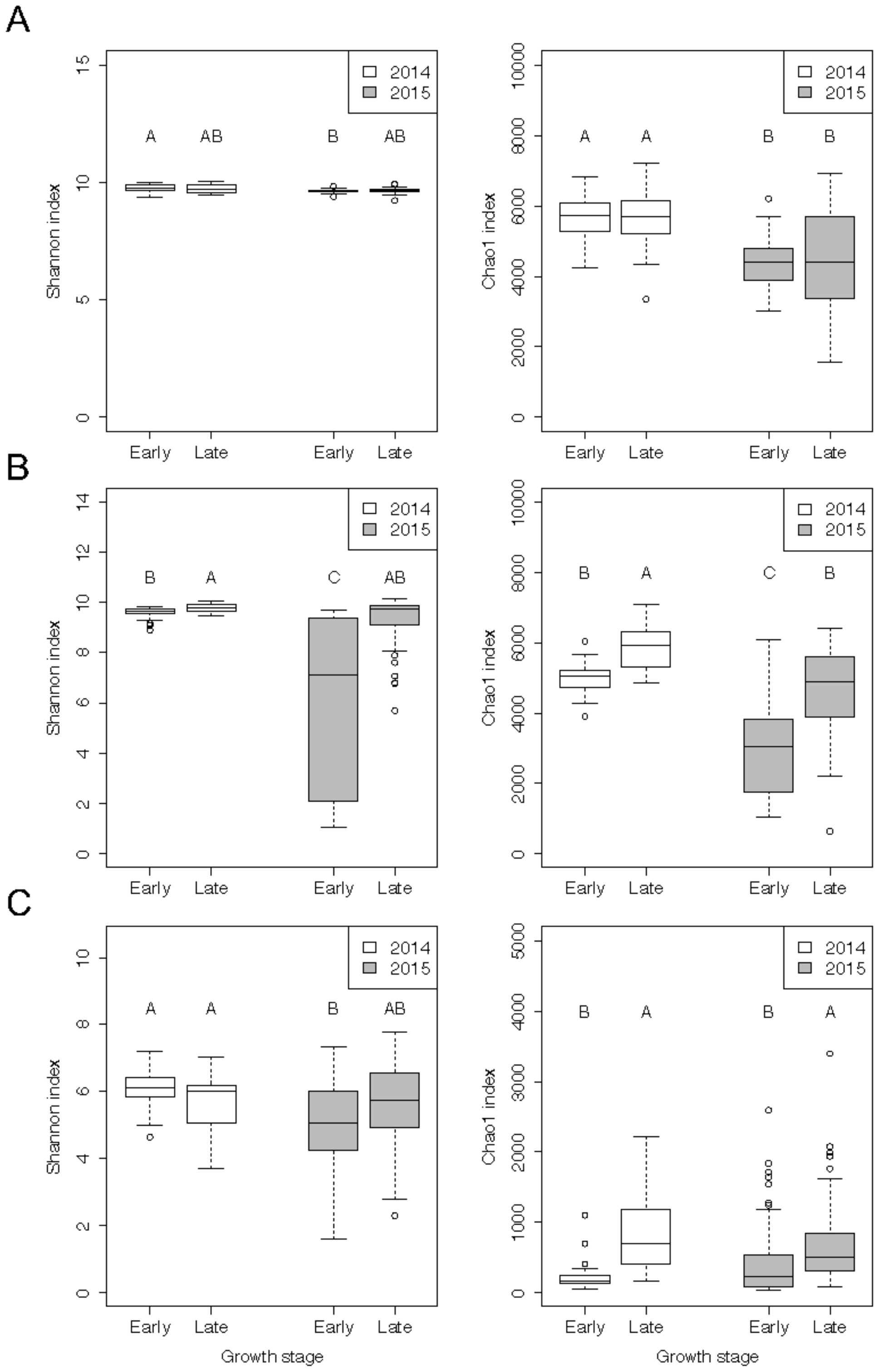

Figure S1. α-diversity differences between years and plant growth stages. Box plots of the species diversity calculated with Shannon index on left and species richness calculated by Chao 1 index on right between the two growth stages and years for the (**A**) soil, (**B**) rhizosphere and (**C**) root endosphere compartments. Distinct letters indicate significant differences according to Wilcoxon's pairwise test.

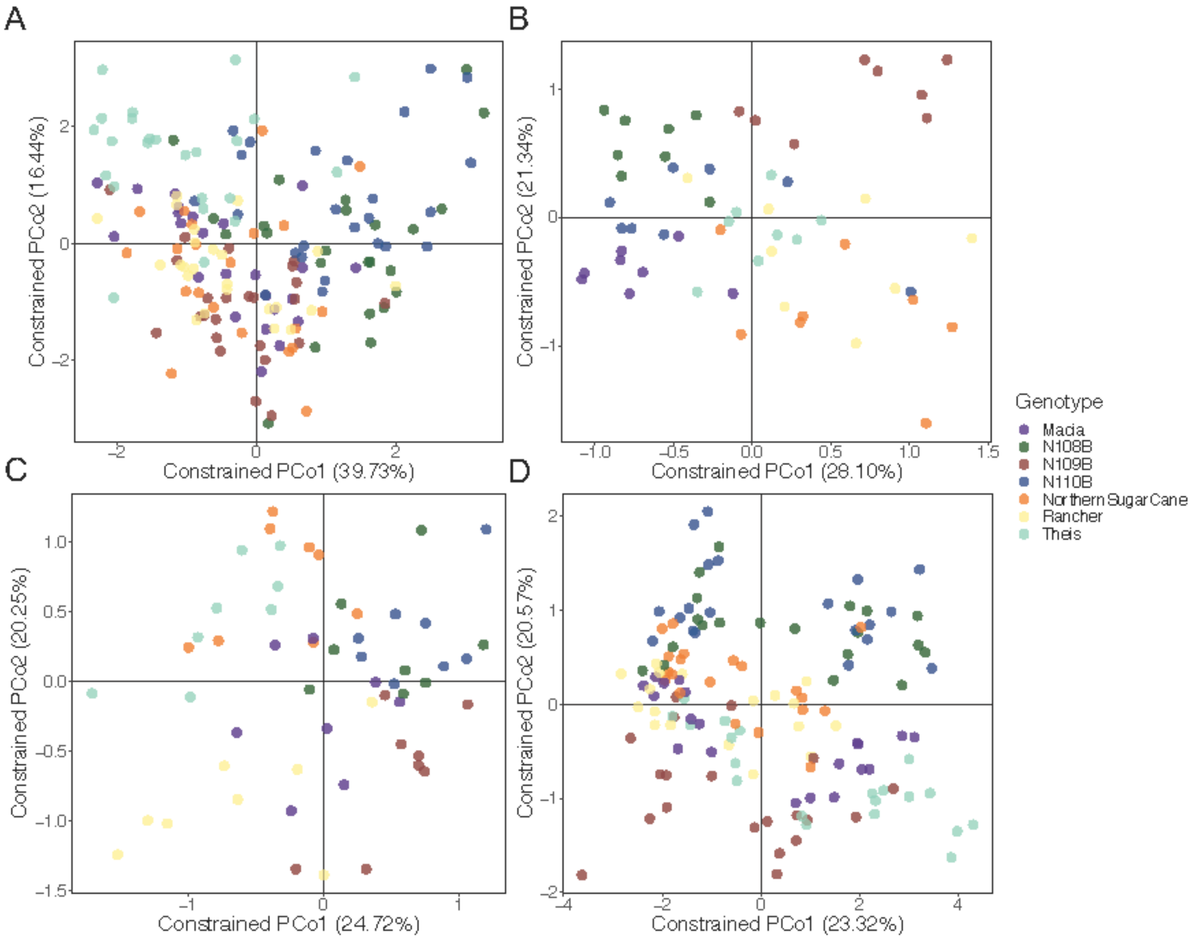

Figure S2. β-diversity differences between sweet sorghum genotypes. Canonical analysis of principal coordinates (CAP) based on the Bray-Curtis distance matrices showing samples of the different sorghum genotypes from (**A**) root endosphere 2014 and 2015, (**B**) soil 2014, (**C**) rhizosphere 2014, and D) rhizosphere 2015. All other factors except genotypes were conditioned in the model.

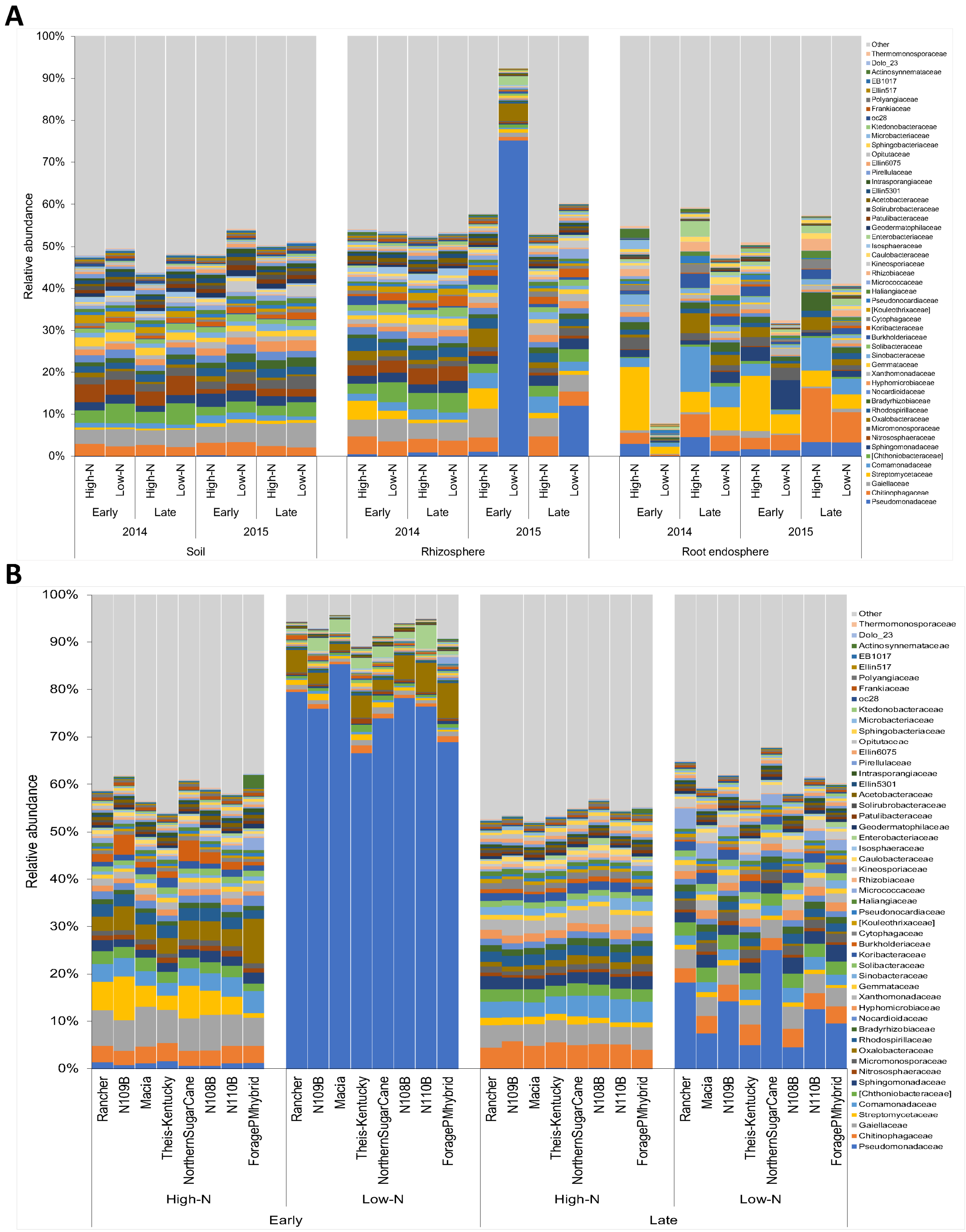

Figure S3. Bar chart showing the relative abundance of bacterial families. (**A**) Relative abundance of the top 50 most abundant bacterial families in the root endosphere, rhizosphere and soil compartments in 2014 and 2015 separated by growth stage and N-level. (**B**) Rhizosphere samples from 2015 highlighting the very high abundance of *Pseudomonas* spp. in all genotypes at the early plant growth stage under low-N. Pseudomonadaceae family is shown in blue bar. The families found in the lowest abundance were combined into "other".

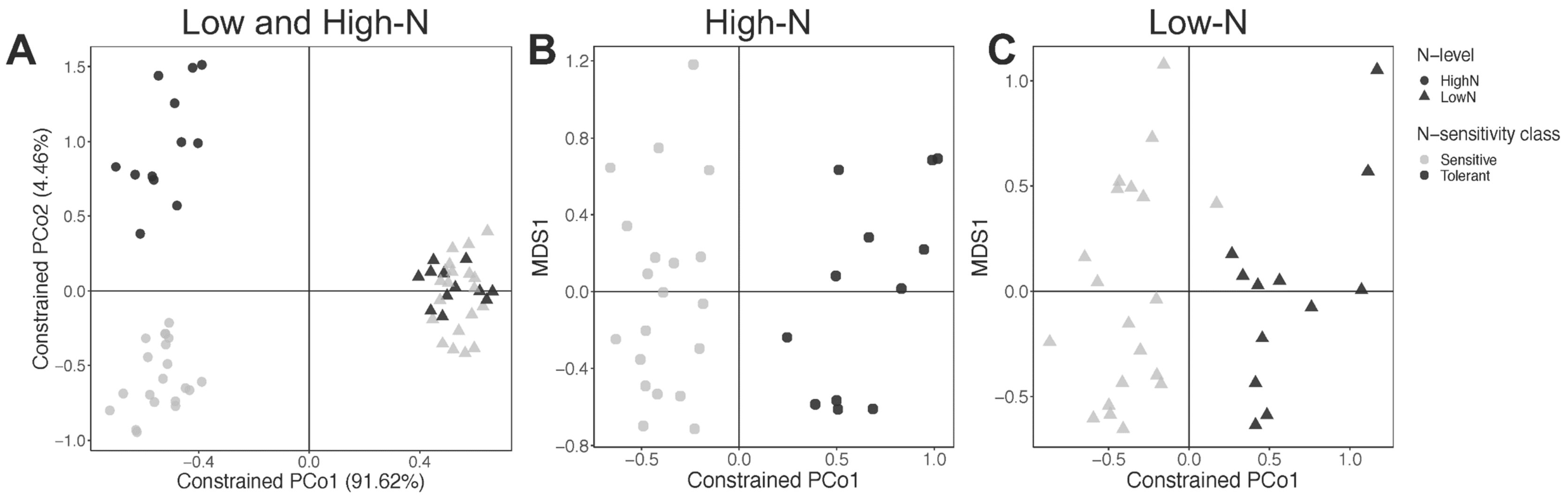

Figure S4. Bacterial community structure of soil samples from N-stress tolerant and sensitive genotypes. Canonical analysis of principal coordinates (CAP) based on the Bray-Curtis distance matrices considering soil samples from (A) both N-levels, (**B**) only the high-N field, and (**C**) only the low-N field. Soil N-levels are represented with different symbols, while sorghum genotype N-stress sensitivity classes are represented with different colors. Only samples from 2014 were used in the analysis, since samples from different genotypes were bulked in 2015.
